# False Identity Detection Using Complex Sentences

**DOI:** 10.3389/fpsyg.2018.00283

**Published:** 2018-03-06

**Authors:** Merylin Monaro, Luciano Gamberini, Francesca Zecchinato, Giuseppe Sartori

**Affiliations:** ^1^Human Inspired Technology Research Centre, University of Padova, Padova, Italy; ^2^Department of General Psychology, University of Padova, Padova, Italy

**Keywords:** lie detection, faked identities, deception detection, complex questions, reaction times

## Abstract

The use of faked identities is a current issue for both physical and online security. In this paper, we test the differences between subjects who report their true identity and the ones who give fake identity responding to control, simple, and complex questions. Asking complex questions is a new procedure for increasing liars' cognitive load, which is presented in this paper for the first time. The experiment consisted in an identity verification task, during which response time and errors were collected. Twenty participants were instructed to lie about their identity, whereas the other 20 were asked to respond truthfully. Different machine learning (ML) models were trained, reaching an accuracy level around 90–95% in distinguishing liars from truth tellers based on error rate and response time. Then, to evaluate the generalization and replicability of these models, a new sample of 10 participants were tested and classified, obtaining an accuracy between 80 and 90%. In short, results indicate that liars may be efficiently distinguished from truth tellers on the basis of their response times and errors to complex questions, with an adequate generalization accuracy of the classification models.

## Introduction

Detecting faked identities is a major issue in security (Barber, 2015), both in the real word and in the internet environment. Concerning the physical security, the National Commission on Terrorist Attacks Upon the United States, that was established after the twin towers terrorist attack on September 11, 2001 in New York, strongly recommended the use of biometric measures to avoid that people traveling under faked identities can cross the international borders (National Commission on Terrorist Attacks Upon the United States, 2004).

However, biometric identification tools currently used (e.g., fingerprints, retinas scan, hand geometry; Ashbourn, 2000) cannot be applied when people are unknown and databases containing their fingerprints are not available. Considering also the online security, the scenario of the fake identities becomes more intricate. The creation of fake accounts and the identity frauds are now really common (Heather, 2012) and dangerous for both a social (e.g., child grooming; Cano et al., 2014) and an economic perspective (e.g., financial crimes, spamming, phishing, etc., Pontell, 2009).

These problems are not currently solved, even if the research is putting a great effort in this direction, also studying new lie detection techniques with possible application in real and online environment (Verschuere and Kleinberg, 2016; Monaro et al., 2017a,c).

Many studies in literature have shown that people who tell lies can be distinguished from those who tell the truth analyzing the features of their responses. For instance, when interrogated, liars give shorter answers than truth tellers (Sporer and Sharman, 2006). This happen for at least two reasons (Vrij et al., 2008a): firstly, they give short answers purposefully in order to don't give the opportunity to the observer to caught them in lying, and secondly because lying is more cognitively demanding then truth telling. Based on this second observation, cognitive-based lie detection techniques have been developed (Vrij, 2015). Cognitive-based lie detection capitalizes on the additional cognitive effort that lying requires compared with truth telling. To date, three main cognitive-based lie techniques have been applied to faked identity detection: the Concealed Information Test (CIT-RT; Verschuere and Kleinberg, 2016), the autobiographical Implicit Association Test (aIAT; Agosta and Sartori, 2013), and the technique of unexpected questions merged with mouse dynamics or keystroke dynamics (Monaro et al., 2017b, 2018). Both the CIT-RT (Verschuere et al., 2011) and aIAT (Sartori et al., 2008) are memory detection techniques. The CIT-RT consists of presenting critical information within a series of noncritical sources of distractor information. In other words, it distinguishes the identities of liars and truth tellers, contrasting the responses to information about the true identity with those to information about the faked identity (Verschuere et al., 2011). The aIAT allows one to verify whether an autobiographical memory is encoded within the mind of the respondent. In other terms, it is able to evaluate which one of two autobiographical events is true and, consequently, to detect the fake event (Agosta and Sartori, 2013). To conclude, CIT-RT and aIAT can identify which between two alternative memories about the identity is the truth and which is the false with high accuracy (from 86 to 94%; Verschuere and Kleinberg, 2016). However, they require that the true identity is available and submitted together with the faked identity to the suspect. In real cases, like terrorists, the actual identity of the suspect is unknown, and this makes impossible to build a CIT or aIAT test, since they required also the true information among the stimuli.

Monaro et al. have overcome the limit of aIAT and CTI thought a new lie detection paradigm based on the subject's response to unexpected questions using mouse or keystroke dynamics (Monaro et al., 2017b, 2018). These identity verification tests do not require knowledge of the suspect's true identity. Liars were instructed to preliminarily overlearn a new identity and to respond as if this faked identity were the true one. Then, both liars and truth tellers were asked to response questions about identity that appeared on the computer screen, by clicking with the mouse on the correct response alternative (e.g., “*yes”* or “*no”*) or typing the response on the computer keyboard. Questions could be expected (e.g., “*were you born in April?”*) or unexpected (e.g., “*is Aries your zodiac sign?”*). Expected questions concerned the information explicitly learned by the liars during the learning phase, such as the date of birth. Unexpected questions referred to information related to those learned during the learning phase, but not explicitly rehearsed, such as the zodiac sign that is an information related to the date of birth. In literature, unexpected questions are questions that focus on aspects that the guilty participant cannot rehearse (Lancaster et al., 2013). Although it is usually observed that a subject takes more time to produce a lie than the truth (Sheridan and Flowers, 2010), it has been also reported that the difference disappears whenever the subject has a training or on those lies (Van Bockstaele et al., 2012). These lies are therefore called expected lies (Vrij, 2015).

Analyzing errors related to unexpected questions and the features of mouse dynamics or keystroke dynamics (mouse trajectory, mouse velocity, response time, writing speed, etc.), Monaro et al. showed that guilty participants faking their personal identities can be identified with an accuracy around 95% (Monaro et al., 2017b, 2018). The authors found that truth tellers were accurate and fast when responding to both expected and unexpected questions. By contrast, liars were much slower and made many more errors when responding to unexpected questions. Furthermore, as the mouse trajectory was recorded, the liars' trajectory clearly deviated from the typical trajectory observed in truth tellers.

Unexpected questions are a powerful tool for uncovering deception (Vrij et al., 2006), but they cannot be used in every condition. When responding to unexpected questions, liars have to process the information in the questions in real time as quickly as possible so that cognitive processing load is combined with time stress in the performance of the task. Within the cognitive load approach to lie detection (Vrij et al., 2008b), one unsolved problem is the identification of a liar when unexpected questions are not available. Typical conditions when unexpected questions cannot be used are in the so-called “lies of omission,” which consist of denying something that did happen (“*I did not do it”* type of lies) (Van Swol and Braun, 2014). For example, if a guilty subject is denying any wrongdoing (in a “*Did you do it?”* type of question), it is difficult to craft an unexpected question that efficiently uncovers his or her deception.

Here, we will present a new technique for detecting lies when unexpected questions cannot be crafted. The technique consists of presenting complex sentences as a substitute for unexpected questions. The cognitive theory of lie production focuses on the fact that lying is cognitively more challenging than truth telling, as liars need to fabricate their truth rather than just automatically retrieving it as in truth telling (Vrij et al., 2008a). Such fabrication requires extensive cognitive effort to avoid contradiction and verifiability (Debey et al., 2014). To respond to a question deceptively and in a credible manner, cognitive resources are required to inhibit the truthful response (Walczyk et al., 2014), to analyse the interlocutor's reactions, to produce the deceptive response and to adapt the behavior according to the lie. Adding additional cognitive load has been shown to be effective in lie detection research, as it results in a critical overload of working memory in an already-overloaded working memory (Vrij et al., 2006). According to Vrij and co-workers, cognitive load induced in liars is particularly disruptive during investigative interviewing, and unexpected questions are a technique for increasing cognitive load, as they require the liar to check whether the response squares in a credible manner with previous statements (Vrij et al., 2009). Other techniques for increasing cognitive load in liars include recalling an event in reverse order (Vrij et al., 2008b) or using interview techniques that require longer answers to questions (Vrij et al., 2007). Both interviewing techniques have been shown to improve investigators' ability to discriminate between liars and truth tellers through a wide range of cues that reflect the liars' cognitive load, such as increased pauses, decreased blinking, decreased hand movements, harder think. Other conditions increase cognitive load in liars. For example, Williams et al. showed in five experiments that increasing the number of response alternatives among which the liar has to choose renders the liar more detectable (Williams et al., 2013). In other words, they stated that when questions involved more than one possible lie response, liars reveal a greater response latency. By contrast, the authors found that the number of alternatives did not significantly affect response times when individuals told the truth. In fact, in real life situations, the subject has to choose one lie in a range of endless possibilities, deciding which the better one is according to the context. The greater number of alternatives requires more cognitive effort by liars who need to monitor the plausibility of more than one information. One way of increasing the number of alternatives is to use complex questions when verification is required on multiple issues. An example of a complex question may be (“*Is your name X and your age Y?”*), whereas an example of a simple question is “*Is your name X*?” In other words, complex questions are questions comprised of two (or more) target information, whereas simple questions contain only one critical information. Liars may respond to simple questions with the same latency as truth tellers, as they can overlearn the response, especially if they have time to practice (Van Bockstaele et al., 2012). We expect that the verification of complex sentences increases cognitive load in liars by increasing the alternatives. Such an increase in the number of alternatives, as Williams et al. showed, is expected to selectively affect liars. In short, increasing the number of alternatives that require scrutiny is expected to enhance the difference between liars and truth tellers in a sentence verification task, and presenting liars with complex sentences in a sentence verification task could be an avenue for evaluating liars when unexpected questions cannot be used (Williams et al., 2013).

We will report here a proof-of-concept experiment aimed to test the differences between subjects who report their true identity and the ones who give fake identity responding to control, simple, and complex questions. The final goal is to obtain a ML classifier based on errors and RT, which is able to identify with high accuracy and generalizability the subjects lying about their identities. We have used lying about identity because we can directly compare the results of the complex questions technique with the results in the same condition of the unexpected questions technique (Monaro et al., 2017b, 2018).

## Method and procedure

### Participants

In the first stage, 40 Italian-native speaker participants (15 males and 25 females) aged between 19 and 26 (*M* = 22.2, *SD* = 1.54; average years of schooling: *M* = 16.6, *SD* = 1.39, where 8 years = primary school, 13 years = high school, 16 years = Bachelor's degree, 18 years = Master's degree) were tested. They were recruited at the Department of Psychology of Padova University, asking them to voluntarily participate to the experiment. Subjects were assigned randomly to the truth teller group (*N* = 20) or to the liar group (*N* = 20). A *t*-test confirms that the two groups were similar in terms of age and schooling (*p* > 0.01), whereas a Chi-squared test (χ^2^) confirms that they similar also for gender (*p* > 0.01). Using the data collected from these first 40 subjects, we trained a machine learning classifiers obtaining a model to sort liars from truth tellers.

To test the generalizability of the model on new data, we also collected an additional group of 10 Italian-speaking participants, which constituted the test group (two males and eight females, average age = 21.9, *SD* = 2.47; average years of schooling: *M* = 15.9, *SD* = 1.45). Subjects were assigned randomly to the truth tellers (*N* = 5) and to the liars (*N* = 5). The two groups were similar for age (*p* > 0.01 in *t*-test), schooling (*p* > 0.01 in *t*-test) and gender whereas (*p* > 0.01 in Chi-squared test).

An independent *t*-test confirms that training (*N* = 40) and test (*N* = 10) groups were similar in terms of age, gender and schooling (*p* > 0.01 both for age, gender, and schooling).

All of the 50 subjects signed the informed consent agreement before the experiment.

### Experimental procedure

Each participant was initially given an envelope containing different instructions for truth tellers and liars. Although instructions for truth tellers consisted of requiring the subjects to fill in a form with their true biographical data, the liars had to learn a new faked identity and, after 10 min, to fill in an empty faked document with their assigned (false) information. The faked identity was provided to the subjects by the experimenter, rather than permit to the subjects to select their own identity. In fact, a self-generated faked identity could have introduced in the experimental procedure uncontrollable variables due to the familiarity of the faked identity (Verschuere and Kleinberg, 2016). One subject could have generated a completely made up identity while another could have used a faked identity he is highly familiar with (e.g., identity of a close friend).

The faked identity that liars were asked to learn consisted of nine information: name, surname, date of birth (day, month, and year), city of birth, province of birth, city of residence, residence address, marital status and occupation. These are typical information reported on an identification (ID) card. The same information were asked to the truth teller when they filled the form with their true biographical data.

All the liars learned the same faked identity according to the gender. In fact, we prepared two fixed identity profiles, one for women and one for men. The experimenter was instructed to change an information on the faked profile only in the case that it matched with the real identity of the participants, according to the information given by the participant before the experiment in the informed consent.

Afterward, both truth tellers and liars had to perform a distracting task (playing Sudoku), and finally, the experiment started. Sentences were presented in the center of the computer screen, and participants were required to classify the presented sentences as true or false in a binary classification task (YES/NO), pressing one of two alternative keys on keyboard. Participants were required to respond as quickly as possible while, at the same time, minimizing the errors. During the task, we recorded reaction times (RT) and errors for each sentence. The ethics committee for psychological research of the University of Padova approved the experimental procedure.

### Stimuli

The presented sentences were of three types (see Table 1): (i) control sentences (*N* = 20; YES = 10, NO = 10), (ii), simple sentences (*N* = 20; YES = 10, NO = 10), and (iii) complex sentences (*N* = 20; YES = 10, NO = 10). Moreover, the task was preceded by 10 training questions to allow the subject familiarization with the task. The complete list of the stimuli is reported in Appendix 1. Stimuli were made-up expressly for this experiment, taking inspiration those reported by Monaro at al. (Monaro et al., 2017b, 2018). Control sentences were sentences to which both truth tellers and liars had to respond truthfully. These sentences were unrelated to identity and referred to the experimental condition. Half of the control sentences required a YES response (e.g., “*I am sitting in front of a computer”*) and half a NO response (e.g., “*I am climbing a mountain”*). Both liars and truth tellers were required to respond truthfully to these control questions. Simple questions were questions related to the identity and containing only one information. Truth tellers responded to sentences regarding their true identities, whereas liars responded as if their false identity (learnt in the training phase) was their true identities. Half of the simple sentences required a YES response (e.g., “*My name is John”*) and half a NO response (e.g., “*My name is Antony”*). For liars, a YES response was a lie, as it corresponded to the faked identity learnt in the preliminary phase. Complex questions (*N* = 20) included two or three information about identity (e.g., “*I am Mary, a 29 years old girl from Venice”*). These complex sentences required a YES response when all of the information was true, whereas they required a NO response when at least one of the pieces of information included in the sentence was false. In other words, participants were asked to respond with YES when the entire sentence was true, and they responded with NO when there was one or more pieces of false information in the sentences. Because a lie is more cognitively demanding, liars would have fewer cognitive resources available to analyse the complex sentences and to produce the correct responses (Baddeley et al., 2014). As result, they would show poor performance on the task in terms of errors and response times.

**Table 1:** Examples of sentences.

**Sentence type**	**Example of question**	**Truth teller expected response**	**Liar expected response**
Control YES *N* = 10	*I am in front of a computer*.	Truth	Truth
Control NO *N* = 10	*I am climbing a mountain*.	Truth	Truth
Simple YES *N* = 10	*I was born on 20th April*.	Truth	Lie
Simple NO *N* = 10	*I was born on 08.15.1990*.	Truth	Truth
Complex YES *N* = 10	*In 1987, I was born in April in Trieste*.	Truth	Lie
Complex NO *N* = 10	*I was born on 20th April 1987 in Ortona*.	Truth	Truth

*Liars have to respond by lying only for simple and complex YES sentences. If the examinee's real date of birth is 15th October, responding YES to “I was born on 20th April” will be a lie*.

For each participant, we averaged the RT and errors belonging to different type of questions (control, simple, complex). We calculated also the Inverse Efficiency Score (IES) for control, simple, and complex questions. IES is an index that combines speed and accuracy (Bruyer and Brysbaert, 2011). Typically, increased response speed is possible but it leads to more errors. This index takes into account the number of errors and increases proportionally the average RT of the subject according to the following formula: RT/(1–PE (Percentage of Error)).

The final list of predictors that have been taken into account for the analysis is reported in Appendix 2.

## Results

### Descriptive statistics

Descriptive statistics are reported for RT and errors, averaged over stimuli for each subject and then averaged over subjects in Table 2. To examine the statistical differences in the collected data between truth tellers and liars, a first analysis was run using R software (“ez anova” package; *https://cran.r-project.org/web/packages/ez/ez.pdf*) (see Additional file 1 for data and Additional file 2 for ANOVA R code; see Additional file 4 for all the raw features data of the 40 training set participants).

**Table 2:** Average RT and errors of liars and truth TELLERS for control, simple, and complex questions.

**RT(M in ms ±*SD*)**	**Total (*N* = 60)**	**Control (*N* = 20)**		**Simple (*N* = 20)**		**Complex (*N* = 20)**	
		**YES**	**NO**	**YES**	**NO**	**YES**	**NO**
LIARS *N* = 20	1,974 ± 321.66	1,491 ± 302.2	1,570 ± 353.14	1,796 ± 328.78	1,748 ± 292.41	2,644 ± 711.76	2,596 ± 580.58
		0.75	0.79	0.90	0.88	1.33	**1.31**
TRUTH TELLERS *N* = 20	1,389 ± 273.36	1,283 ± 266.74	1,251 ± 0246.41	1,201 ± 337	1,238 ± 270.18	1,842 ± 396.82	1,521 ± 286.72
		0.92	0.90	0.86	0.89	1.32	**1.10**
**Errors (M** ± **SD**)	**Total (*N* = 6 0)**	**Control (*N* = 20)**		**Simple (*N* = 20)**		**Complex (*N* = 20)**	
		**YES**	**NO**	**YES**	**NO**	**YES**	**NO**
LIARS *N* = 20	0.093 ± 0.092	0.05 ± 0.20	0.065 ± 0.22	0.10 ± 0.10	0.10 ± 0.10	0.08 ± 0.13	0.165 ± 0.19
		0.53	0.69	1.07	1.07	0.86	1.77
TRUTH TELLERS *N* = 20	0.014 ± 0.013	0.015 ± 0.05	0.005 ± 0.02	0.005 ± 0.02	0.01 ± 0.03	0.025 ± 0.05	0.025 ± 0.04
		1.07	0.35	0.35	0.71	1.78	1.78

*The table reports the average of RT (in ms) and the average number of errors (calculated as the ratio between the number of errors and the number of stimuli) for the two experimental groups (liars and truth tellers). SD are also reported (±). Under RT, the ratio between the RT and the overall RT is reported. What is clear is that although liars, in responding with NO to complex sentences, are 30% slower that the average RT (first column), truth tellers in the same stimuli are only 10% slower*.

**Figure 1:**
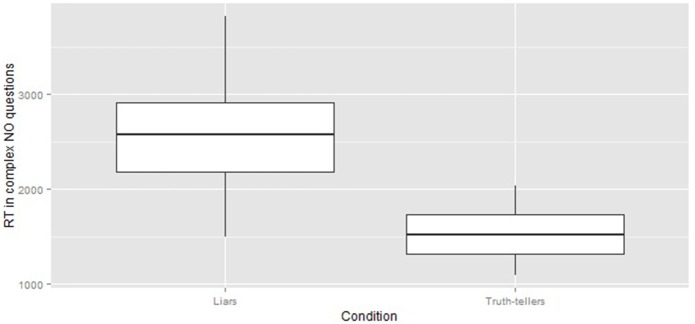
The box plots compare the RT of liars and truth tellers in complex questions that required a NO response.

An ANOVA indicated that:

- overall, the responses of liars were longer than those of truth tellers [*F*_(1, 38)_ = 38.39 *p* < 0.001]. Figure 1 compares RT of liars and truth tellers in complex NO questions;
- complex questions were slower than simple questions for both liars and truth tellers [*F*_(2, 76)_ = 147.45 *p* < 0.001], but complex sentences were much slower, with respect to simple sentences, in liars than in truth tellers [*F*_(2, 76)_ = 25.22, *p* < 0.01]. In fact, the difference in RT between complex and simple sentences was 848 ms for liars and only 463 ms for truth tellers;
- there is not a main effect of the response type (yes/no) [*F*_(1, 38)_ = 2.34, *p* > 0.01]. The interactions group X response type, question type X response type and group X question type X response type do not show statistically significant results (respectively [*F*_(1, 38)_ = 1.88, *p* > 0.01], [*F*_(2, 76)_ = 4.62, *p* > 0.01] and [*F*_(2, 76)_ = 2.91, *p* > 0.01]). It means that generally, both liars and truth tellers, have the same RTs when responding yes or no questions. It excludes the possibility that the effect observed in the complex sentences is due to the act of negating rather than the lie itself.

It is worth noting that the number of errors that liars made, on average, was 5.6 times the number of errors of truth tellers.

### Feature selection

The analysis reported here were conducted using the machine learning software WEKA 3.8 (Hall et al., 2009). Wide consensus exists on the fact that in developing machine learning (ML) models for classification, the preliminary identification of non-redundant features (predictors) is an important step in the development of an effective predictive model which maximizes generalization (Hall, 1998). Non-redundant features are those which have a high correlation with the dependent variable (truth teller vs. liar) while having a low intercorrelation among them. To understand better this point, it could be useful to observe the correlation matrix between all the variables that is provided in Additional file 6. Independent variables which entered the feature selection are those listed in Appendix 2. The non-redundant features have been extracted using a correlation based feature selector (CFS; Hall, 1998). This type of algorithm evaluates the worth of a subset of features by considering the individual predictive ability of each feature along with the degree of redundancy with the other predictors. Subsets of features that are highly correlated with the class (the dependent variable) while having low intercorrelation are preferred. There are different methods that the algorithm can use to search the subset of predictors through the spaces of features. Here, a Greedy Stepwise search method has been used. It performs a greedy forward or backward search (in this case, a forward search has been used) through the space of predictors subsets. It may start with no/all attributes or from an arbitrary point in the space (here it started with no attributes). It stops when the addition/deletion of any remaining attributes results in a decrease in evaluation. Through running this algorithm, the following predictors were identified: (1) Simple Yes RT r_pb_ = 0.67, (2) Complex Tot RT r_pb_ = 0.73, (3) Complex No RT r_pb_ = 0.77, (4) Mean Total errors r_pb_ = 0.55 and (5) Mean Simple Tot errors r_pb_ = 0.66, where r_pb_ is the correlation value of the predictor with the dependent variable.

### Machine learning classification

We developed a number of ML models to evaluate the classification accuracy. The results reported below have been collected using the 10-fold cross-validation technique. Cross-validation is a technique used to evaluate predictive models by partitioning the original sample (in this case, one of 40 participants) into two subset of data: a subset called training set, that is used to build the predictive model, and a subset called validation set that is used to evaluate the model built on the training set. In 10-fold cross-validation, the original sample is randomly partitioned into 10 equal-size subsamples. Of the 10 subsamples, a single subsample was retained as the validation data for testing the model, and the remaining 10-1 subsamples were used as training data. The cross-validation process was then repeated 10 times (the folds), with each of the 10 subsamples used exactly once as the validation data. The 10 final results from the folds were then averaged to produce a single estimation of prediction accuracy. The advantage of this method is that all observations are used for both training and validation, and each observation is used for validation exactly once.

We evaluated the prediction accuracy using five different ML models to evaluate whether the results were stable across classifiers and did not depend on the specific assumptions that each of the models makes. In fact, the five classifiers used are representative of differing underlying classification strategy. Logistic measures the relationship between the categorical dependent variable and the independent variables by estimating probabilities using a logistic function (le Cessie and van Houwelingen, 1992). Support Vector Machine (SVM) is a non-probabilistic binary linear classifier, which maps the space and divide the examples of the separate categories by a clear gap that is as wide as possible (Platt, 1999; Keerthi et al., 2001). Naïve Bayes is a probabilistic classifier based on Bayes' theorem that assumes the independence between the features (John and Langley, 1995). Random Forest (Breiman, 2001) operates by constructing a multitude of decision trees and Logistic Model Tree (LMT) combines logistic regression and decision tree learning (Landwehr et al., 2005). Moreover, to understand better the decision rules on which the classifications results are based on, we ran a tree classification model J48 (Quinlan, 1993). It is one of the simplest—if not the simplest—classifier in terms transparency of the operations computed by the algorithm and it permits to easily highlighting the classification logic (even if not the most efficient; Mitchell, 1997). In other words, it is helpful to explain the operations performed by the algorithm on the data to obtain the classification output.

Default parameters were used in order to minimize overfitting (for all the details on ML classifiers parameters see Additional file 3).

The results indicated that all classifiers classified the 40 participants as liars or truth tellers with at least 90% accuracy. Specifically: Logistic = 90%, Support Vector Machine (SVM) = 95%, Naïve Bayes = 90%, Random Forest = 90%, LMT = 95% and J48 = 85%.

To compare the performance of the different classifiers, we have run an experiment in WEKA (Hall et al., 2009) using the paired *t*-tester. The results showed that none of pairwise algorithm comparisons reached the significance level (*p* > 0.05), indicating that the classifiers have a comparable accuracy.

To check the generalization of the models developed using the 10-fold cross-validation, we used the second sample of 10 test participants previously described (see Additional file 5 for the raw features data of the 10 test set participants; see Additional file 3 for the instructions to replicate classification results on training and test set). The results obtained in this group of 10 participants were the following: Logistic = 80%, SMO (SVM) = 80%, Naïve Bayes = 90%, Random Forest = 80%, LMT = 80% and J48 = 90%. Despite a slight reduction in the overall accuracy, the generalization accuracy should be considered adequate.

**Figure 2:**
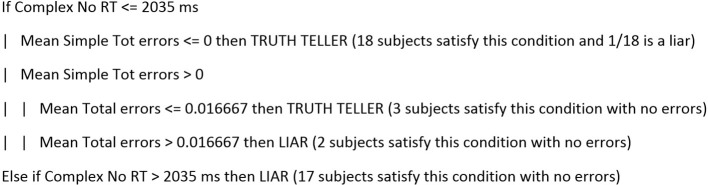
This decision tree translated into words indicates that truth tellers are those subjects who have an average RT to complex NO responses below 2,035 ms and make no errors. If the RT is below 2,035 ms and the subject makes errors in responding to simple sentences but the average total number of errors is still below 0.01, then he or she is a truth teller. By contrast, if the average total number of errors is above 0.02 or the RT in the complex NO sentences is above 2,035 ms, the responder is a liar.

ML models, such as some of those reported above, are difficult to interpret. It is not clear the mechanics that yield the classifier to identify the single participant as a liar or truth teller. However, other ML models may shed light on which predictors mostly contribute to an adequate classification. One such model is a pruned tree model called J48 (Quinlan, 1993). A decision tree has a number of advantages; specifically: (i) they are easy to understand; (ii) they are easily converted to a set of production rules; and (iii) no a priori assumptions about the nature of the data exist. This model, when run on the group of 40 subjects, yielded an accuracy of 85% and an accuracy of 90% for the classification of the 10 participants of the test group. The decision tree (J48) classification criteria are reported in Figure 2.

The analysis reported above was conducted on raw data using two groups of participants (liars and truth tellers) who were similar in age, cultural level, and typing skills. One could argue that the RT results are modulated by a number of different variables, such as age, cultural level, etc. To render the results generalizable, it would be interesting to see whether similar results hold true not only for raw data but also for normalized predictors. Under this view, raw RT for YES responses could be substituted by the ratio of the same data with the average RT of all of the subject responses. This ratio calibrates the result with the average speed of the participant, which, in turn, could depend on a number of factors. For this reason, we ran again the classification models using only normalized predictors, which are supposed to be less vulnerable to inter-individual and environmental variables. The complete list of the normalized predictors is provided in Appendix 2.

The same attribute selection procedure reported previously highlighted a subset of normalized predictors (the r_pb_ number refers to the correlation of the independent variable with the group). The predictors are the following: (1) Control Yes RT/Total RT r_pb_ = 0.59, (2) Complex Tot RT/Total RT r_pb_ = 0.56, (3) Complex No RT/Total RT r_pb_ = 0.66, (4) (Complex Yes RT–Complex No RT)/Total RT r_pb_ = 0.39 and (5) Raw Simple Tot errors/Raw Control Tot errors r_pb_ = 0.60. Similarly, the results of the 40 participants were the following: (1) Logistic = 85%, (2) SVM = 87.5%, (3) Naïve Bayes = 90%, (4) Random Forest = 90%, (5) LMT = 85% and (6) J48 = 77.5%. Again, a paired *t*-test confirms that the six classifiers have a comparable accuracy (*p* > 0.5).

The results of the classifiers applied to the 10 subjects' test sample were the following: (1) Logistic = 90%, (2) SVM = 80%, (3) Naïve Bayes = 80%, (4) Random Forest = 90%, (5) LMT = 90% and (6) J48 = 90%. In short, when using normalized predictors, we observed similar results to those observed on raw data. The adoption of such normalized predictors instead of raw data renders, in theory, generalization more robust and less affected by the effects on RT of age, skill level, etc.

### Analysis by stimuli

The results reported above were obtained with an analysis by subjects, and therefore, the accuracy of classifiers refers to the accuracy in classifying individual responders as liars or truth tellers.

An interesting issue is whether the subject may be classified based on his/her individual responses through a majority vote. Given that the responses to complex sentences which require a NO response are those which show a higher correlation with the group (liar vs. truth teller), we carried out the classification by stimuli using only these responses. A total of 400 responses were collected (40 subjects who responded each to 10 sentences which required a NO response, for a total of 400 sentences). The predictors were the RT to the presented sentence and a categorical variable indexing whether the response was correct or wrong. Using these, two independent variables, the results from the 10-fold cross validation were the following: (1) Logistic = 75.5%, (2) SVM = 73%, (3) Naïve Bayes = 73.5%, (4) Random Forest = 69.25%, (5) LMT = 74.5% and (6) J48 = 73%.

A pair *t*-test shows that all the classifiers have comparable accuracies (*p* > 0.05), except for Random Forest and J48 that have a significantly different performance (*p* < 0.05) with lower scores.

The generalization level of these classifiers was evaluated on a total of 100 new sentences (complex NO) derived from the 10 participants of the test sample (10 participants responding to 10 sentences each). The results were the following: (1) Logistic = 74%, (2) SVM = 70%, (3) Naïve Bayes = 69%, (4) Random Forest = 70%,(5) LMT and (6) J48 = 72%. The results may be summarized as follows: each of the 10 responses that a participant gave to a Complex NO sentence may be identified as originating from a truth teller or a liar with 70% accuracy. Using the majority rule to classify a participant as a truth teller or liar, the 10 participants of the validation sample were classified correctly 80% of the time (all truth tellers were correctly classified, whereas two liars were misclassified). An intuition on how a participant may be efficiently classified could be derived from tree classification model J48 (described above), which yielded an accuracy of 73% on the 400 stimuli. Such a result was replicated in the 100 stimuli collected from the 10 subjects of the test sample (overall accuracy = 72%). The decision tree, J48, that gave the previous results was the following:

- If RT < = 1870 ms and errors = 0, then the responder is a truth teller (with accuracy = 75%).
- If RT < = 1870 ms and errors = 1, the responder is a truth teller (with accuracy = 70%).
- If RT > 1870 ms, then the responder is a liar (with accuracy = 78%).

In short, if RT is fast (below 1870 ms) and the response correct, then the responder is a truth teller.

If RT is slow (above 1870 ms), then the responder is a liar. Finally, if RT is fast but the response is an error, the truth teller is classified as having a slightly reduced accuracy.

## Discussion and conclusion

Cognitive load has been shown to be an effective tool for identifying liars during investigative interviewing. In addition, cognitive load has been achieved with a number of differing techniques (unexpected questions, drawing, dual tasking, recounting events in reverse order, keep eyes contact, etc., Walczyk et al., 2013; Vrij et al., 2017).

We previously showed that unexpected questions efficiently identify liars about personal identity (Monaro et al., 2017b, 2018). Suppose that an examinee is lying about his date of birth, reporting (and overlearning) a false one. A related unexpected question directly linked to the date of birth is “*Which is your zodiac?*” Although a truth teller has the information readily available, the liar has to compute it on the spot, and this different strategy reflects itself in a longer RT and a higher error rate. However, on some occasions, unexpected questions may not be used, leaving open the problem of detecting liars under such conditions. Unexpected questions cannot be used when the liar is simply denying (“*I did not do it”* types of questions), as these types of questions are not suited for deriving unexpected questions.

In fact, when a lie is expected and overlearned, the typical response retardation that characterizes a lie disappears (Hu et al., 2012).

Here, we are presenting a technique for increasing cognitive load to detect liars that does not rely on unexpected questions. Cognitive load is increased by requiring participants to verify complex sentences. A complex sentence is “*I was born in Rome and I live in Venice*.” Such complex sentences may require a YES response if all of the information is correct, and they will require a NO response when at least one piece of information is wrong. Complex sentences are intermixed in the experiment with simple sentences about identity (e.g., “*I was born in Rome”*) and with control questions (e.g., “*I am sitted on a chair”*). Here, we have reported a proof-of-concept experiment using the complex questions technique for identifying respondents who lie about their true identities. Complex sentences, in a YES/NO verification task, require more cognitive load. We have confirmed this hypothesis by finding that, for both liars and truth tellers, complex sentences have longer RT than do simple sentences. However, this difference is much larger in liars. Liars have responses to complex NO sentences which are 30% longer than their overall RT. By contrast, the same figure for truth tellers is 10%. Given that the same pattern is replicated for errors, we can conclude that liars have more problems with responding to complex sentences than do truth tellers. This difference can be used to distinguish liars from truth tellers with levels of accuracy approaching the one observed in a lie detection technique based on the use of unexpected questions (Monaro et al., 2017b, 2018). The classification accuracy achieved using raw data was above 90% with no bias between false positives and false negatives. To obtain this result, we choose to analyzed data through ML algorithms that gave different classification models as output. ML is now very popular in data science community and it is used to make reliable predictions or classifications, with the advantage to automate the outcome for new sample of data. In fact, it enables to train one or more algorithms to predict outcomes without being explicitly programmed and only uses the information learned from the training set. Moreover, ML models usually outperform traditional statistical models.

One important issue in behavioral research is the reproducibility of results and their generalization to a group of subjects different from the one used to develop the model (Dwork et al., 2015; Open Science Collaboration, 2015). Generalization and replicability are particularly problematic for ML models, and we have addressed this issue using the following strategies:

1. Development of five ML models which have diverse underlying assumptions. Stability of results across classifiers is an indication that results are not dependent on specific assumptions.
2. A test group of 10 participants was collected after the models were developed in the original group of 40 participants. The performance on this test sample is intended to maximize the replicability of the results to new subjects.
3. Analysis were carried out both by subjects (averaging over stimuli) and by stimuli. Both analysis indicate good generalization to new subjects and new stimuli.

ML models are considered very efficient in classification accuracy, but most of them are opaque, as the mechanics of classification are highly complex and avoid intuition. For this reason, we developed a decision tree model, which may shed light on this issue. The results of this interpretable model may be summarized as follows:

- liars may be efficiently distinguished from truth tellers on the basis of their response times and errors most specifically with regard to complex sentences which require a NO response;
- liars are slow when responding to complex NO sentences and make 5.6 times the number of errors that truth tellers make;
- truth tellers are fast and make no errors when responding to complex NO sentences.

In short, when unexpected questions are unavailable, the analysis of RT and error rates for complex questions (especially complex NO questions) efficiently spots participants with faked identities, with an accuracy comparable to that obtained using unexpected questions.

Given the enhancement of the number of the terrorists using false identities (Barber, 2015) and the strong connection between online frauds and identity theft (Pontell, 2009), this study represents a step forward to address the issue related to the detection of faked identities. We think that the final goal of the research advancement should be to create an identity-screening tool for large-scale applications, as the migration flow control or the online identity verification. Following this direction, the methodology that we propose in this paper seems to be promising. It has shown a level of accuracy enough to be apply as a screening instrument, to identify people who require further consideration from the border patrol or identify the illegitimate users of an internet account. The main advantage of this methodology, unlike other cognitive deception detection methods, such as CIT and aIAT, is that it does not require any a priori information (e.g., the real identity of the suspect) to get the truth. Therefore, it is suitable for all those cases in which the investigator has simply to establish if a declaration given by the suspect is truthful or not.

However, the experimental paradigm that we used to test the subjects suffers from some limitations. First, subjects were instructed to lie about their identity, so they did not lie spontaneously. Secondly, the participants' motivation and the effort to lie are different from the real situation. To overcome these limitations, the experiment should be replicated in an ecological environment, where subjects are encouraged to lie spontaneously and to put their effort in the task driven by a real motivation. Moreover, future researches should be focused to investigate the generalizability of the methodology to other forensic topic, such as alibi verification.

## Ethics statement

The ethics committee for psychological research of the University of Padova approved the experimental procedure (Unique Number: C93848566A4018338F947BD887B505FA). All of the subjects who were involved in the study signed the informed consent agreement before the experiment.

### Availability of data and materials

The dataset used and analyzed during the current study is available in Supplementary Material.

## Author contributions

MM and GS: Conceived the experiment; MM and FZ: Designed the experimental task and acquired data; MM and GS: Analyzed and interpreted the data. All of the authors were involved in drafting the manuscript. GS and LG: Revised the manuscript critically and gave the final approval of the version to be published.

### Conflict of interest statement

The authors declare that the research was conducted in the absence of any commercial or financial relationships that could be construed as a potential conflict of interest.

## Supplementary material

Additional file 1 ANOVA data. Data Description: the file contains the RT raw data of the 40 training set participants to compute ANOVA using ANOVA R code. (.txt 10 kb)

Additional file 2 ANOVA R code. File description: the file contains the R code used to compute ANOVA on data in Additional file 1. (.txt 1 kb).

Additional file 3 Instructions to replicate correlation analysis, features selection and classification results. File Description: the file contains the step by step instructions to replicate the results of correlation analysis, features selection and machine learning classification in training set and test set. (.pdf 230 kb).

Additional file 4 Training set data. Data Description: the file contains the data (raw features) of the 40 training set participants. (.xlsx 15 kb).

Additional file 5 Test set data. Data Description: the file contains the data (raw features) of the 10 test set participants. (.xlsx 10 kb).

Additional file 6 Correlation matrix between the predictors and between the predictors and the dependent variable.

## Abbreviations

aIAT: autobiographical Implicit Association Test
CIT: Concealed Information Test
IES: Inverse Efficiency Score
LMT: Logistic Model Tree
ML: Machine Learning
PE: Percentage of Errors
RT: Reaction Times
SMO: Sequential Minimal Optimization
SVM: Support Vector Machine.
