# Supplementary material for: False Identity Detection Using Complex Sentences
Source: Front Psychol. 2018 Mar 6;9:283. doi: 10.3389/fpsyg.2018.00283 (PMC5845552; doi:10.3389/fpsyg.2018.00283)
Supplement: Additional file 1 — ANOVA data. Data Description: the file contains the RT raw data of the 40 training set participants to compute ANOVA using ANOVA R code. (.txt 10 kb) [file DataSheet1.zip › Additional files/Additional file 3.pdf]

Additional file 3. Instructions to replicate correlation analysis, features selection and classification results.

Machine Learning (ML) Models were implemented using WEKA software Version 3.8.

The software can be free downloaded at this link: http://www.cs.waikato.ac.nz/ml/weka/downloading.html . A complete documentation about WEKA software is available at this link: http://www.cs.waikato.ac.nz/ml/weka/ .

Classification results that are reported in the paper were obtained following these steps:

1) Open WEKA 3.8 and choose the *Explorer* application.
2) From the *Preprocess Panel* open the file "**Additional file 4**" (data from 40 participants). This file contains the data of the raw features that the authors used in the paper to classify subjects as liars or truth-tellers (see Appendix 2 in the manuscript). The variable named "Condition" is the class to predict (truth-teller vs liar).
3) To perform the correlation analysis switch in *Select Attributes Panel* and choose "CorrelationAttributeEval" as Attribute Evaluator, "Ranker" as Search Method and "Use full training set" as Attribute Selection Mode. Click on "Start" to visualize the correlation analysis output.
4) To perform the features selection remain in *Select Attributes Panel* and choose "CfsSubsetEval" as Attribute Evaluator, "GreedyStepwise" as Search Method and "Use full training set" as Attribute Selection Mode. Click on "Start" to visualize the attribute selection output.
5) Switch in *Process Panel* and remove from the Attribute list all the attributes except the attributes selected in the previous step (attributes 8,10,12,13,15) and the class to predict ("Condition").
6) Switch in *Classify Panel* and choose Cross-validation as *Test option* setting the number of folds to 10.
7) To replicate the paper results, from the *Classifier* list choose Logistic, SMO, NaïveBayes, Random Forest, LMT, J48 and run the models. The analyses were carried out using the default parameters of classifiers. The details on the default ML classifiers parameters are reported in **Annex 1**.
8) To test the 5 model obtained by the 10-fold cross-validation on the test sample (10 new participants), select Supplied test set as *Test option*. Upload the file "**Additional file 5**" as supplied test set. Then, to replicate the paper results on test sample, choose Random Forest, Logistic, SMO, Naïve Bayes, LMT, J48 from the *Classifier* list and run the models. The analyses were carried out using the default parameters of classifiers. The details on the default ML classifiers parameters are reported in **Annex 1**.

Annex 1 - Details on ML classifiers parameters

The analyses were carried out in WEKA (Version 3.8) using the default parameters. Note that there is no fine tuning of the parameters in order to increase the classification accuracy.

Random Forest:
weka.classifiers.trees.RandomForest -P 100 -I 100 -num-slots 1 -K 0 -M 1.0 -V 0.001 -S 1

- seed -- The random number seed to be used. = 1
- storeOutOfBagPredictions -- Whether to store the out-of-bag predictions. = FALSE
- numExecutionSlots -- The number of execution slots (threads) to use for constructing the ensemble. = 1
- bagSizePercent -- Size of each bag, as a percentage of the training set size. = 100
- numDecimalPlaces -- The number of decimal places to be used for the output of numbers in the model. = 2
- batchSize -- The preferred number of instances to process if batch prediction is being performed. More or fewer instances may be provided, but this gives implementations a chance to specify a preferred batch size. = 100
- printClassifiers -- Print the individual classifiers in the output. = FALSE
- numIterations -- The number of iterations to be performed. = 100
- debug -- If set to true, classifier may output additional info to the console. = FALSE
- outputOutOfBagComplexityStatistics -- Whether to output complexity-based statistics when out-of-bag evaluation is performed. = FALSE
- breakTiesRandomly -- Break ties randomly when several attributes look equally good. = FALSE
- doNotCheckCapabilities -- If set, classifier capabilities are not checked before classifier is built (Use with caution to reduce runtime). = FALSE
- maxDepth -- The maximum depth of the tree, 0 for unlimited. = 0
- calcOutOfBag -- Whether the out-of-bag error is calculated. = FALSE
- numFeatures -- Sets the number of randomly chosen attributes. If 0, int(log_2(#predictors) + 1) is used. = 0

Logistic:
weka.classifiers.functions.Logistic -R 1.0E-8 -M -1 -num-decimal-places 4

- numDecimalPlaces -- The number of decimal places to be used for the output of numbers in the model. = 4
- batchSize -- The preferred number of instances to process if batch prediction is being performed. More or fewer instances may be provided, but this gives implementations a chance to specify a preferred batch size. = 100
- debug -- Output debug information to the console. = FALSE
- ridge -- Set the Ridge value in the log-likelihood. = 1.0E-8
- useConjugateGradientDescent -- Use conjugate gradient descent rather than BFGS updates; faster for problems with many parameters. = FALSE
- maxIts -- Maximum number of iterations to perform. = -1
- doNotCheckCapabilities -- If set, classifier capabilities are not checked before classifier is built (Use with caution to reduce runtime). = FALSE

SMO:
weka.classifiers.functions.SMO -C 1.0 -L 0.001 -P 1.0E-12 -N 0 -V -1 -W 1 -K "weka.classifiers.functions.supportVector.PolyKernel -E 1.0 -C 250007" -calibrator "weka.classifiers.functions.Logistic -R 1.0E-8 -M -1 -num-decimal-places 4"

- buildCalibrationModels -- Whether to fit calibration models to the SVM's outputs (for proper probability estimates). = FALSE
- numFolds -- The number of folds for cross-validation used to generate training data for calibration models (-1 means use training data). = -1
- randomSeed -- Random number seed for the cross-validation. = 1
- c -- The complexity parameter C. = 1.0
- numDecimalPlaces -- The number of decimal places to be used for the output of numbers in the model. = 2
- batchSize -- The preferred number of instances to process if batch prediction is being performed. More or fewer instances may be provided, but this gives implementations a chance to specify a preferred batch size. = 100
- kernel -- The kernel to use. Polykernel –C 250007 -E 1.0
- checksTurnedOff -- Turns time-consuming checks off - use with caution. = FALSE
- debug -- If set to true, classifier may output additional info to the console. = FALSE
- filterType -- Determines how/if the data will be transformed. = Normalized training data
- toleranceParameter -- The tolerance parameter (shouldn't be changed). = 0.001
- calibrator -- The calibration method to use. = Logistic
- doNotCheckCapabilities -- If set, classifier capabilities are not checked before classifier is built (Use with caution to reduce runtime). = FALSE
- epsilon -- The epsilon for round-off error (shouldn't be changed). 1.0E-12

LMT:
weka.classifiers.trees.LMT -I -1 -M 15 -W 0.0
- splitOnResiduals -- Set splitting criterion based on the residuals of LogitBoost. There are two possible splitting criteria for LMT: the default is to use the C4.5 splitting criterion that uses information gain on the class variable. The other splitting criterion tries to improve the purity in the residuals produces when fitting the logistic regression functions. The choice of the splitting criterion does not usually affect classification accuracy much, but can produce different trees. = FALSE
- useAIC -- The AIC is used to determine when to stop LogitBoost iterations. The default is not to use AIC. = FALSE
- numDecimalPlaces -- The number of decimal places to be used for the output of coefficients. = 2
- batchSize -- The preferred number of instances to process if batch prediction is being performed. More or fewer instances may be provided, but this gives implementations a chance to specify a preferred batch size. = 100
- weightTrimBeta -- Set the beta value used for weight trimming in LogitBoost. Only instances carrying (1 - beta)% of the weight from previous iteration are used in the next iteration. Set to 0 for no weight trimming. The default value is 0. = 0.0
- doNotMakeSplitPointActualValue -- If true, the split point is not relocated to an actual data value. This can yield substantial speed-ups for large datasets with numeric attributes. = FALSE
- debug -- If set to true, classifier may output additional info to the console. = FALSE
- numBoostingIterations -- Set a fixed number of iterations for LogitBoost. If >= 0, this sets a fixed number of LogitBoost iterations that is used everywhere in the tree. If < 0, the number is cross-validated. = -1
- fastRegression -- Use heuristic that avoids cross-validating the number of Logit-Boost iterations at every node. When fitting the logistic regression functions at a node, LMT has to determine the number of LogitBoost iterations to run. Originally, this number was cross-validated at every node in the tree. To save time, this heuristic cross-validates the number only

once and then uses that number at every node in the tree. Usually this does not decrease accuracy but improves runtime considerably. = TRUE

- minNumInstances -- Set the minimum number of instances at which a node is considered for splitting. The default value is 15. = 15
- doNotCheckCapabilities -- If set, classifier capabilities are not checked before classifier is built (Use with caution to reduce runtime). = FALSE
- errorOnProbabilities -- Minimize error on probabilities instead of misclassification error when cross-validating the number of LogitBoost iterations. When set, the number of LogitBoost iterations is chosen that minimizes the root mean squared error instead of the misclassification error. = FALSE
- convertNominal -- Convert all nominal attributes to binary ones before building the tree. This means that all splits in the final tree will be binary. = FALSE

NAÏVE BAYES:
weka.classifiers.bayes.NaiveBayes

- useKernelEstimator -- Use a kernel estimator for numeric attributes rather than a normal distribution. = FALSE
- numDecimalPlaces -- The number of decimal places to be used for the output of numbers in the model. = 2
- batchSize -- The preferred number of instances to process if batch prediction is being performed. More or fewer instances may be provided, but this gives implementations a chance to specify a preferred batch size. = 100
- debug -- If set to true, classifier may output additional info to the console. = FALSE
- displayModelInOldFormat -- Use old format for model output. The old format is better when there are many class values. The new format is better when there are fewer classes and many attributes. = FALSE
- doNotCheckCapabilities -- If set, classifier capabilities are not checked before classifier is built (Use with caution to reduce runtime). = FALSE
- useSupervisedDiscretization -- Use supervised discretization to convert numeric attributes to nominal ones. = FALSE

J48:
weka.classifiers.trees.J48

- batchSize -- The preferred number of instances to process if batch prediction is being performed. More or fewer instances may be provided, but this gives implementations a chance to specify a preferred batch size. = 100
- binarySplits -- Whether to use binary splits on nominal attributes when building the trees. = FALSE
- collapseTree -- Whether parts are removed that do not reduce training error. = TRUE
- confidenceFactor -- The confidence factor used for pruning (smaller values incur more pruning). = 0.25
- debug -- If set to true, classifier may output additional info to the console. = FALSE
- doNotCheckCapabilities -- If set, classifier capabilities are not checked before classifier is built (Use with caution to reduce runtime). = FALSE
- doNotMakeSplitPointActualValue -- If true, the split point is not relocated to an actual data value. This can yield substantial speed-ups for large datasets with numeric attributes. = FALSE
- minNumObj -- The minimum number of instances per leaf. = 2

- numDecimalPlaces -- The number of decimal places to be used for the output of numbers in the model. = 2
- numFolds -- Determines the amount of data used for reduced-error pruning. One fold is used for pruning, the rest for growing the tree. = 3
- reducedErrorPruning -- Whether reduced-error pruning is used instead of C.4.5 pruning. = FALSE
- saveInstanceData -- Whether to save the training data for visualization. = FALSE
- seed -- The seed used for randomizing the data when reduced-error pruning is used. = 1
- subtreeRaising -- Whether to consider the subtree raising operation when pruning. = TRUE
- unpruned -- Whether pruning is performed. = FALSE
- useLaplace -- Whether counts at leaves are smoothed based on Laplace. = FALSE
- useMDLcorrection -- Whether MDL correction is used when finding splits on numeric attributes. = TRUE
