# Supplementary material for: False Identity Detection Using Complex Sentences
Source: Front Psychol. 2018 Mar 6;9:283. doi: 10.3389/fpsyg.2018.00283 (PMC5845552; doi:10.3389/fpsyg.2018.00283)
Supplement: Supplementary file 2 [file Presentation1.pdf]

APPENDIX 1

Sentences used in the experiment (N=60)

<u>CONTROL TRUE</u> (CONTROL YES, N=10)

I am in front of a computer.

I am standing in front of a computer.

I'm using a computer.

I am responding with a keyboard.

Now, I'm sitting on a chair.

Right now, I'm sitting.

I'm doing an identity verification test.

I am participating in a test.

I'm reading sentences.

I am responding to phrases.

<u>CONTROL FALSE</u> (CONTROL NO, N=10)

I'm swimming in the sea.

I'm climbing a mountain.

I'm traveling by airplane.

I am aboard an airplane.

Now, I am on the beach.

I'm taking in the sun at the beach.

I'm eating at the restaurant.

I'm having lunch at the restaurant.

I'm playing football.

I'm watching a football match.

10 SIMPLE TRUE (SIMPLE YES, N=10)

Liars who respond with a faked identity have to respond with TRUE based on the profile they overlearned in a preliminary phase of the experiment.

My name is Merylin.

I was born in Trieste.

I live in Monfalcone.

I am single.

My last name is Monaro.

I am a student.

I was born in the province of Trieste (for the United States [US], this could be: I was born in California State).

I live in Via Timavo 47.

I was born on 20th April.

I was born in 1987.

10 SIMPLE FALSE (SIMPLE NO, N=10)

My last name is Zurri.

My name is Greta.

I was born on 08.15.1990.

My birthday is in August.

I'm married.

My city of birth is Ortona.

I live in Lanciano.

I am an engineer.

I was born in the province of Chieti (for the US, this could be: I was born in Florida State).

I live in via Postojna.

10 COMPLEX TRUE (FIVE WITH TWO INFO, AND FIVE WITH THREE INFO) (COMPLEX YES, N=10)

I am Merylin, born in Trieste.

My name is Merylin Monaro.

I was born in Trieste, and I live in Monfalcone.

I was born in April 1987 in Trieste.

I am a student, and I live in Via Timavo 47.

In the 1987, I was born in April in Trieste.

I am Monaro, a single student.

My name is Merylin, I am single and I live in Monfalcone.

I am the student Merylin, born on 20.04.1987.

I am the student Merylin, born in April.

10 COMPLEX FALSE (FIVE WITH TWO INFO (THE **FALSE** INFO IS ALWAYS IN THE SECOND PLACE), FIVE WITH THREE INFO (THREE WITH ONLY ONE **FALSE** INFO IN THE LAST PLACE AND TWO WITH TWO **FALSE** INFO IN THE SECOND AND THIRD PLACES) (COMPLEX NO, N=10)

I am Merylin, and I live in **Lanciano**.

I was born on 20th April 1987 in **Ortona**.

I was born in Trieste, and I live in **Lanciano**.

I am a student, and I am **married**.

I live in Monfalcone in **via Postojna 65**.

I am Merylin, a single **engineer**.

I am Merylin, a student born in **August**.

Merylin Monaro was born in **August 1990**.

I am Merylin **Zurri**, and I am **married**.

I am Monaro, a **married** woman of **Ortona**.

APPENDIX 2

Raw predictors	
**Total RT**	Mean Reaction Time on all test questions
**Total Yes RT**	Mean Reaction Time on all test questions that required a YES response
**Total No RT**	Mean Reaction Time on all test questions that required a NO response
**Control Tot RT**	Mean Reaction Time on all control questions
**Control Yes RT**	Mean Reaction Time on control questions that required a YES response
**Control No RT**	Mean Reaction Time on control questions that required a NO response
**Simple Tot RT**	Mean Reaction Time on all simple questions
**Simple Yes RT**	Mean Reaction Time on simple questions that required a YES response
**Simple No RT**	Mean Reaction Time on simple questions that required a NO response
**Complex Tot RT**	Mean Reaction Time on all complex questions
**Complex Yes RT**	Mean Reaction Time on complex questions that required a YES response
**Complex No RT**	Mean Reaction Time on complex questions that required a NO response
**Mean Total errors**	Mean number of errors on all test questions (number of errors on all test questions divided by total number of stimuli on entire test)
**Mean Control Tot errors**	Mean number of errors on all control questions (number of errors on all control questions divided by total number of control stimuli)
**Mean Simple Tot errors**	Mean number of errors on all simple questions (number of errors on all simple questions divided by total number of simple stimuli)
**Mean Complex Tot errors**	Mean number of errors on all complex questions (number of errors on all complex questions divided by total number of complex stimuli)
**Raw Total errors**	Total number of errors on all test questions
**Raw Control Tot errors**	Number of errors on all control questions
**Raw Simple Tot errors**	Number of errors on all simple questions

**Raw Complex Tot errors**	Number of errors on all complex questions
**IES Control**	Inverse Efficiency Score calculated for RT and errors of control questions
**IES Simple**	Inverse Efficiency Score calculated for RT and errors of simple questions
**IES Complex**	Inverse Efficiency Score calculated for RT and errors of complex questions
**Normalized predictors**	
**Total Yes RT/Total RT**	Ratio between RT on all test questions that required a YES response and RT on all test questions
**Total No RT/Total RT**	Ratio between RT on all test questions that required a NO response and RT on all test questions
**Control Tot RT/Total RT**	Ratio between RT on all control questions and RT on all test questions
**Control Yes RT/Total RT**	Ratio between RT on control questions that required a YES response and RT on all test questions
**Control No RT/Total RT**	Ratio between RT on control questions that required a NO response and RT on all test questions
**Simple Tot RT/Total RT**	Ratio between RT on all simple questions and RT on all test questions
**Simple Yes RT/Total RT**	Ratio between RT on simple questions that required a YES response and RT on all test questions
**Simple No RT/Total RT**	Ratio between RT on simple questions that required a NO response and RT on all test questions
**Complex Tot RT/Total RT**	Ratio between RT on all complex questions and RT on all test questions
**Complex Yes RT/Total RT**	Ratio between RT on complex questions that required a YES response and RT on all test questions

**Complex No RT/Total RT**	Ratio between RT on complex questions that required a NO response and RT on all test questions
**(Total Yes RT-Total No RT)/Total RT**	Ratio of the difference between the RT on all test questions that required a YES response and the RT on all test questions that required a NO response with the RT on all test questions
**(Control Yes RT - Control No RT)/Total RT**	Ratio of the difference between the RT on control questions that required a YES response and the RT on control questions that required a NO response with the RT on all test questions
**(Simple Yes RT - Simple No RT)/Total RT**	Ratio of the difference between the RT on simple questions that required a YES response and the RT on simple questions that required a NO response with the RT on all test questions
**(Complex Yes RT - Complex No RT)/Total RT**	Ratio of the difference between the RT on complex questions that required a YES response and the RT on complex questions that required a NO response with the RT on all test questions
**Raw Simple Tot errors/Raw Control Tot errors**	Ratio between the number of errors on all simple questions and the number of errors on all control questions
**Raw Complex Tot errors/Raw Control Tot errors**	Ratio between the number of errors on all complex questions and the number of errors on all control questions
**Raw Complex Tot errors/Raw Simple Tot errors**	Ratio between the number of errors on all complex questions and the number of errors on all simple questions
